# RETRACTION: Assessment of Cognitive–Motor Functions in Adults With Perceived Neuropsychological Problems Using NIH Toolbox After Remote Biofield Energy Treatment as Non‐Pharmacological Intervention: A Randomized Double‐Blind Placebo Controlled Trial

**DOI:** 10.1002/npr2.70098

**Published:** 2026-02-17

## Abstract

**RETRACTION**: TrivediM. K. , BrantonA. , TrivediD. , MondalS. and JanaS. , “Assessment of Cognitive–Motor Functions in Adults With Perceived Neuropsychological Problems Using NIH Toolbox After Remote Biofield Energy Treatment as Non‐Pharmacological Intervention: A Randomized Double‐Blind Placebo Controlled Trial,” Neuropsychopharmacology Reports 44, no. 4 (2024): 749‐761, 10.1002/npr2.12482.39270308 PMC11609756

The above article, published online on 13 September 2024 in Wiley Online Library (wileyonlinelibrary.com), has been retracted by agreement between the journal Editor‐in‐Chief, Tsuyoshi Miyakawa; the Japanese Society of Neuropsychopharmacology; and John Wiley & Sons Australia, Ltd. The retraction has been agreed upon as the study's design, methods, results and conclusions are essentially the same as another article published elsewhere by the same author group in the same year, without any attribution to that article. Furthermore, the study contains physiologically implausible data and statistical anomalies. The editors consider the results and conclusions of this article to be invalid. The authors do not agree with the retraction.

**RETRACTION**: M. K. Trivedi , A. Branton , D. Trivedi , S. Mondal and S. Jana , “Assessment of Cognitive–Motor Functions in Adults With Perceived Neuropsychological Problems Using NIH Toolbox After Remote Biofield Energy Treatment as Non‐Pharmacological Intervention: A Randomized Double‐Blind Placebo Controlled Trial,” Neuropsychopharmacology Reports 44, no. 4 (2024): 749‐761, 10.1002/npr2.12482.39270308 PMC11609756

The above article, published online on 13 September 2024 in Wiley Online Library (wileyonlinelibrary.com), has been retracted by agreement between the journal Editor‐in‐Chief, Tsuyoshi Miyakawa; the Japanese Society of Neuropsychopharmacology; and John Wiley & Sons Australia, Ltd. The retraction has been agreed upon as the study's design, methods, results and conclusions are essentially the same as another article published elsewhere by the same author group in the same year, without any attribution to that article. Furthermore, the study contains physiologically implausible data and statistical anomalies. The editors consider the results and conclusions of this article to be invalid. The authors do not agree with the retraction.

